# Optimizing the Ratio of One-Off Slow-Release Fertilizer Can Improve the Nitrogen Use Efficiency and Yield of Rice Under the Condition of Nitrogen Reduction

**DOI:** 10.3390/plants14233650

**Published:** 2025-11-29

**Authors:** Zichen Liu, Zilin Wang, Gaoyuan Wu, Junlei Chen, Jingqi He, Meikang Wu, Dongchao Wang, Xiaoshuang Wei, Ping Tian, Zhihai Wu, Siyuan Li, Meiying Yang

**Affiliations:** 1 Faculty of Agronomy, Jilin Agricultural University, Changchun 130118, China; l3307185039@163.com (Z.L.); 20240809@mails.jlau.edu.cn (Z.W.); gy15034700516@163.com (G.W.); 20210076@mails.jlau.edu.cn (J.C.); he13578512755@163.com (J.H.); wumeikang@mails.jlau.edu.cn (M.W.); dongchaowang@mails.jlau.edu.cn (D.W.); weixiaoshuang@jlau.edu.cn (X.W.); tianping@jlau.edu.cn (P.T.); wuzhihai@jlau.edu.cn (Z.W.); 2 Jilin Province Green and High Quality Japonica Rice Engineering Research Center, Changchun 130118, China; 3 College of Life Sciences, Jilin Agricultural University, Changchun 130118, China

**Keywords:** Nitrogen fertilizer, slow-release fertilizer ratio, yield, nitrogen use efficiency, photosynthetic rate

## Abstract

Increasing grain production is crucial for national food security, and fertilizer management is one of the most effective ways to achieve this. In traditional agricultural production, excessive nitrogen (N) application often leads to reduced N use efficiency and increased environmental pollution. Compound slow-release fertilizers can effectively improve N use efficiency while still meeting the nutritional demands of rice. However, research on these compound slow-release fertilizers remains limited. The effects of fertilizer management measures (controlled-release fertilizer ratios and N fertilizer levels) on rice yield, material accumulation, photosynthetic characteristics, and N use efficiency are not yet fully understood. In particular, the relationships between yield and material accumulation, photosynthetic characteristics, and N use efficiency require further study. Therefore, this study was conducted in 2021 and 2022 using Jinongda 667 as the material, with three N fertilizer rates of 90 (N1), 120 (N2), and 150 kg ha^−1^ N (N3). Six controlled-release fertilizer ratios of sulfur-coated urea (SCU) and resin-coated urea (RCU) were tested: 1:0 (C1), 0:1 (C2), 3:1 (C3), 4:1 (C4), 5:1 (C5), and 6:1 (C6) (optimized in 2022 to three ratios: 3:1, 4:1, and 5:1, with traditional split-application fertilization (CF) added as a control). The results showed that the N3C5 treatment achieved the highest yield of 9246.7 kg ha^−1^ among all combinations of slow-controlled release compound fertilizer ratios and N levels. In 2021, under the same N gradient, yields followed the order C5 > C4 > C3 > C2 > C1, whereas the C6 treatment exhibited a declining yield trend across different N levels. The yield pattern observed in 2022 was consistent with that of 2021. Further comparisons of C3, C4, and C5 under different N levels with the traditional fertilization treatment (N3CF) indicated that, under the same N level, the C5 treatment produced significantly higher yields than the C3 and C4 treatments. Photosynthetic rates at various stages under the C5 treatment increased by 1.9% to 12.7% compared to the C3 and C4 treatments. The N2C5 and N3C5 treatments increased yield by 1.3% and 9.4%, respectively, compared with N3CF, with effective panicle numbers increasing by 6.7% and 11.1%, respectively. The N2C5 treatment reduced N application by 20% compared with N3CF, while significantly increasing N Apparent Use Efficiency (NAUE) by 56.6% and Agronomic N Agricultural Utilization Efficiency (NAE) by 41.8%. Therefore, applying a 5:1 controlled-release fertilizer at a N application rate of 120 kg ha^−1^ can reduce N use while enhancing efficiency. This approach provides a theoretical basis for green, high-yield rice cultivation.

## 1. Introduction

Rice (*Oryza sativa* L.) is a staple crop in China. Against the backdrop of a growing population, the continued growth of rice production is essential for national food security [1]. N fertilizer, a major input in rice cultivation, plays a crucial role in boosting grain yields to meet the rising demand for rice [2,3]. However, excessive application of N fertilizers not only results in low NUE but also causes serious environmental pollution, including soil acidification, water eutrophication, increased greenhouse gas emissions, and reduced rice grain quality. To achieve high-yield, efficient rice production, improved N fertilizer management is necessary. Key approaches include precise quantitative cultivation [4], field-specific N management [5], and integrated soil-crop system management [6]. However, due to limited mechanization in rice production, split fertilizer applications increase labor requirements and production costs. As a result, these N management practices are time-intensive and labor-demanding. For example, Peng et al. [7] recommended applying N fertilizer in 3–4 split applications, an approach that does not align with the efficiency demands of modern rice production systems. Compared with conventional urea fertilization, coated controlled-release N fertilizers can overcome these limitations. By providing controlled N release aligned with plant demand [8,9], this technology reduces labor inputs while enhancing nutrient-use efficiency. Controlled-release fertilizers exhibit a release rate that more closely aligns with plant nutritional requirements. Liu et al. [10] demonstrated that combining these fertilizers with conventional urea increases the leaf area index (LAI) in rice. Yang et al. [11] found that rice plants treated with controlled-release urea showed significantly higher leaf SPAD values (indicating relative chlorophyll content), greater total plant N content, and higher soil ammonium and nitrate concentrations from heading to maturity stages compared to conventional urea treatments. Moreover, studies report that controlled-release urea improves crop yield and NUE while simultaneously reducing greenhouse gas emissions and nitrate leaching [12].

Based on production processes, controlled-release fertilizers can be divided into two main categories. One is condensation products of urea and urea-aldehydes (slow-release fertilizers) and the other is coated or encapsulated fertilizers (controlled-release fertilizers) [13]. Sulfur is an essential element for N metabolism and protein synthesis, serving as a key component in enzymatic systems and metabolic processes. It is also particularly vital for plant defense systems against biotic and abiotic stresses [14]. Compared with conventional urea, sulfur-coated urea application significantly enhances crop growth, development, and yield [15]. SCU promotes root growth by increasing root diameter and length, thereby enhancing nutrient uptake [16] and facilitating dry matter assimilation [17]. However, despite its cost-effectiveness, the integrity of the SCU coating has high variability. This inconsistency may cause rapid N release, leading to seedling burn, or conversely, excessively slow release that delays plant development and reduces yields [18]. The main obstacle to adopting resin coatings is their higher production cost compared to traditional N fertilizers. This limits their widespread use in farmland, except for certain high-value crops and nursery plants [19]. Compared with SCU, polymer-coated urea, such as RCU, releases N more slowly. With the continuous supply of N, especially during the later growth stages, it can significantly increase aboveground biomass and grain yield [20].

Xing et al. [21] reported that a mixture of SCU and RCU increased rice yield, accompanied by a larger leaf area index and a higher photosynthetic potential compared with applying either fertilizer alone. However, previous studies on slow- and controlled-release fertilizers have mainly focused on a single type of fertilizer. Few studies have investigated composite slow- and controlled-release fertilizers, particularly the optimal ratios between them. We hypothesized that optimizing the ratio of slow- and controlled-release fertilizers would enhance N use efficiency while maintaining high rice yield. This approach could thereby achieve a synergy among high yield, high N efficiency, and environmental sustainability. Therefore, a two-year field trial was conducted to investigate the effects of N application rates and controlled-release fertilizer ratios on rice yield, photosynthetic capacity, and NUE. Based on a comprehensive analysis of yield components, photosynthetic parameters, and N efficiency indices, an integrated fertilization strategy was developed to achieve high yield, high N efficiency, and environmental sustainability. This study aims to provide theoretical support for optimizing high-yield and high-efficiency rice cultivation. It also seeks to facilitate the development of green rice varieties and improve cultivation techniques.

## 2. Results

### 2.1. Grain Yield

**Figure 1:**
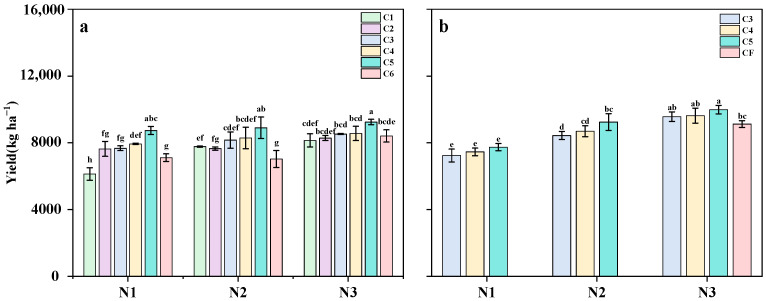
Yield under Different N Application Rates and Controlled-Release Fertilizer Ratios in 2021 (**a**) and 2022 (**b**). Note: Data are means of replicates, and the vertical bars represent the standard error. Different letters above the columns indicate statistical significance at the *p* < 0.05 level for different treatments. N1, 90 kg N ha^−1^; N2, 120 kg N ha^−1^; N3, 150 kg N ha^−1^; C1, sulfur-coated urea (SCU): resin-coated urea (RCU) = 1:0; C2, SCU: RCU = 0:1; C3, SCU: RCU = 3:1; C4, SCU: RCU = 4:1; C5, SCU: RCU = 5:1; C6, SCU: RCU = 6:1; CF, traditional split-application fertilization.

As shown in Figure 1, yield increased with higher N application rates, and this trend was consistent across the two years. For the different controlled-release fertilizer formulations, in 2021, yields generally followed the trend of C5 > C4 > C3 > C2 > C1 (except for the C1 and C2 treatments under N2). Yields from the C6 treatment were consistently lower than those from the C5 treatment. The trends observed in 2022 mirrored those of 2021. Compared with the N3CF treatment, yields of the N2C5 and N3C5 treatments increased by 1.3% and 9.4%, respectively, with N3C5 achieving the highest yield in both years.

### 2.2. Yield Components

**Figure 2:**
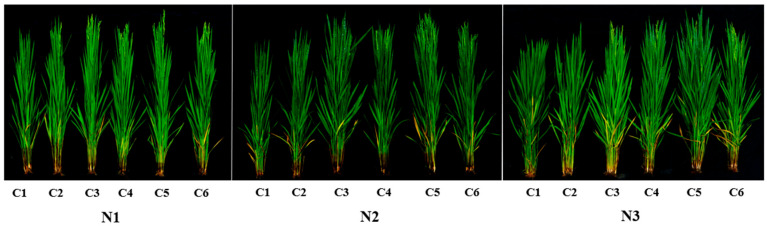
Growth of aboveground parts of rice under different N application rates and different slow and controlled release fertilizer ratios at heading stage in 2021. Note: N1, 90 kg N ha^−1^; N2, 120 kg N ha^−1^; N3, 150 kg N ha^−1^; C1, sulfur-coated urea (SCU): resin-coated urea (RCU) = 1:0; C2, SCU: RCU = 0:1; C3, SCU: RCU = 3:1; C4, SCU: RCU = 4:1; C5, SCU: RCU = 5:1; C6, SCU: RCU = 6:1.

The number of grains per panicle and the number of effective panicles (excluding 2022 data for effective panicles) responded consistently to both N fertilizer and controlled-release fertilizer ratio treatments. These traits increased with higher N fertilizer application and as the controlled-release fertilizer ratio increased from 1:0 to 5:1 but declined at 6:1. Yield differences resulting from N fertilizer were primarily due to variations in effective panicle number and grains per panicle (Table 1 and Table 2). Compared to the N1 treatment, the N2 and N3 treatments increased effective panicle number by 10.1% and 21.2%, respectively, and grains per panicle by 3.1% and 8.7%, respectively. Regarding controlled-release fertilizer ratios, as the ratio of SCU to RCU increased from 3:1 to 5:1, both effective panicle number and grains per panicle over two years followed the trend of C5 > C4 > C3 (except for the number of effective panicles in 2022). However, when the SCU:RCU ratio reached 6:1 in 2021, the number of effective panicles and grains per panicle showed a declining trend. The field growth in rice under the C5 treatment was significantly higher than that in the C4 and C3 treatments at the N3 level (Figure 2). The number of effective panicles in the N2C5 and N3C5 treatments increased by 6.7% and 11.1%, respectively, compared to the N3CF treatment.

### 2.3. Dry Matter Accumulation

**Figure 3:**
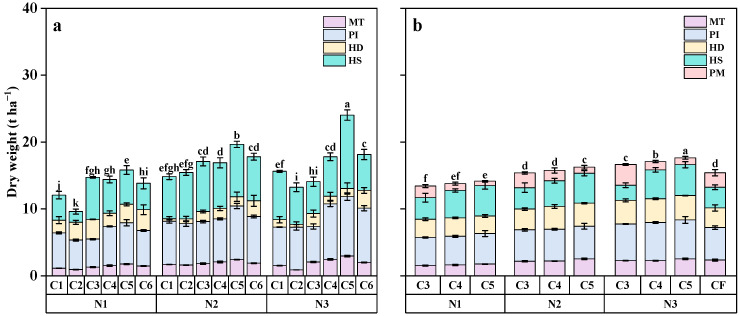
Effect of different slow-release fertilizer ratios on rice dry matter accumulation under different N applications in 2021 (**a**) and 2022 (**b**). Note: Data are means of replicates, and the vertical bars represent the standard error. Different letters above the columns indicate statistical significance at the *p* < 0.05 level for different treatments. N1, 90 kg N ha^−1^; N2, 120 kg N ha^−1^; N3, 150 kg N ha^−1^; C1, sulfur-coated urea (SCU): resin-coated urea (RCU) = 1:0; C2, SCU: RCU = 0:1; C3, SCU: RCU = 3:1; C4, SCU: RCU = 4:1; C5, SCU: RCU = 5:1; C6, SCU: RCU = 6:1; MT, mid-tillering stage; PI, panicle initiation stage; HD, heading stage; FS, filling stage; PM, physiogical maturity stage.

Dry matter accumulation at the Mid-Tillering (MT), Panicle Initiation (PI), Heading (HD), Filling (FS), and Physiological Maturity (PM) stages all increased with higher N application rates. As the ratio of SCU to RCU increased from 3:1 to 5:1, dry matter accumulation also increased with the increase in the proportion of SCU. However, when the ratio reached 6:1, dry matter accumulation began to decline (Figure 3). During the FS stage, average dry matter accumulation under the N2 and N3 treatments was 15 and 16.2 t ha^−1^, respectively, representing increases of 15.0% and 24.1% compared to the N1 treatment (13 t ha^−1^). Compared with the C3 treatment, dry matter accumulation under the C4 and C5 treatments increased by 4.8% and 17.7%, respectively.

### 2.4. Photosynthetic Rate

**Figure 4:**
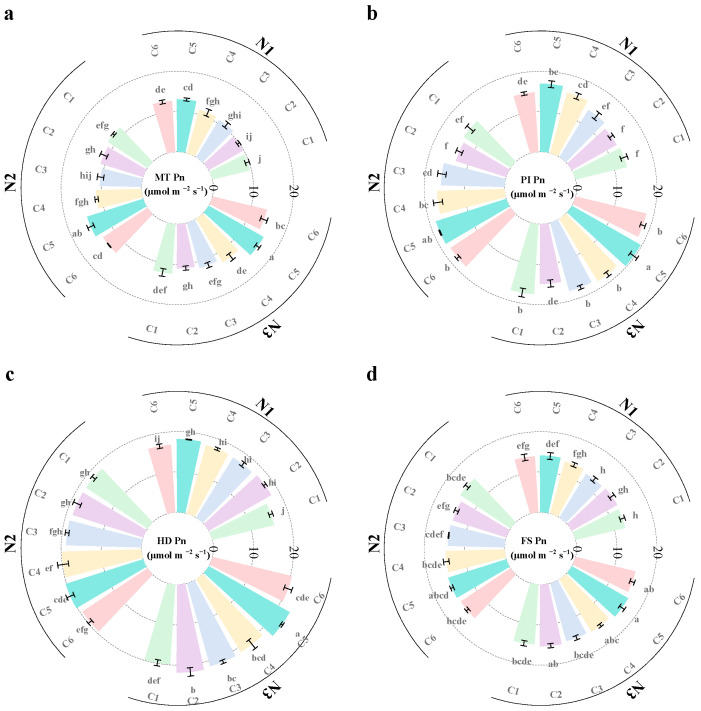
Effects of different slow-release fertilizer ratios at different N application rates on photosynthetic rate at MT (**a**), PI (**b**), HD (**c**) and FS (**d**) of rice in 2021. Note: Data are means of replicates, and the vertical bars represent the standard error. Different letters above the columns indicate statistical significance at the *p* < 0.05 level for different treatments. N1, 90 kg N ha^−1^; N2, 120 kg N ha^−1^; N3, 150 kg N ha^−1^; C1, sulfur-coated urea (SCU): resin-coated urea (RCU) = 1:0; C2, SCU: RCU = 0:1; C3, SCU: RCU = 3:1; C4, SCU: RCU = 4:1; C5, SCU: RCU = 5:1; C6, SCU: RCU = 6:1.

**Figure 5:**
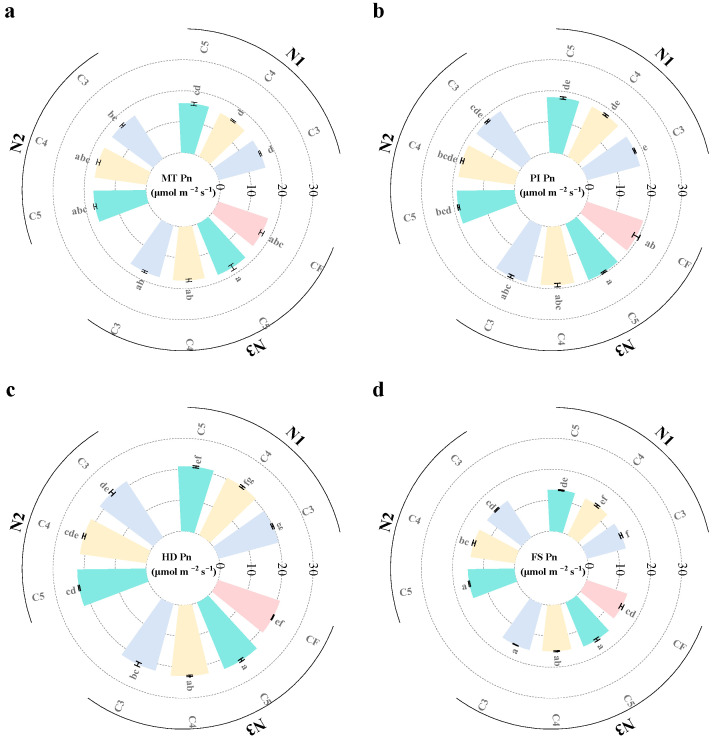
Effects of different slow-release fertilizer ratios at different N application rates on photosynthetic rate at MT (**a**), PI (**b**), HD (**c**) and FS (**d**) of rice in 2022. Note: Data are means of replicates, and the vertical bars represent the standard error. Different letters above the columns indicate statistical significance at the *p* < 0.05 level for different treatments. N1, 90 kg N ha^−1^; N2, 120 kg N ha^−1^; N3, 150 kg N ha^−1^; C3, sulfur-coated urea (SCU): resin-coated urea (RCU) = 3:1; C4, SCU: RCU = 4:1; C5, SCU: RCU = 5:1; CF, traditional split-application fertilization.

Both N fertilizer application and controlled-release fertilizer application significantly impact the net photosynthetic rate of rice. Overall, the net photosynthetic rate of rice tends to increase initially and then decrease as the growth stage progresses. Photosynthetic rates at different growth stages increased with higher N application rates (Figure 4 and Figure 5). Compared with the N1 treatment, the N2 treatment increased photosynthetic rates by 7.5%, 7%, 8.6%, and 10.3% during the MT, PI, HD, and FS stages, respectively. The N3 treatment increased photosynthetic rates by 13.3%, 14.8%, 17.3%, and 15.7% at the same stages. In terms of the ratio of controlled-release fertilizers, the photosynthetic rates of rice under the single application of SCU (C1) and RCU (C2) treatments were lower than those under the combined application of both fertilizers. As the SCU-to-RCU ratio increased from 3:1 to 5:1, the photosynthetic rate showed an upward trend. Under the C4 treatment, the photosynthetic rates at each stage were 3.9%, 3.8%, 1.9%, and 2.0% higher than those under the C3 treatment, respectively. Under the C5 treatment, the photosynthetic rates at each stage were 12.7%, 5.3%, 3.5%, and 4.0% higher than those under the C3 treatment, respectively. However, when the SCU:RCU ratio increased from 5:1 to 6:1, the photosynthetic rate exhibited a decreasing trend. Overall, the net photosynthetic rates at all growth stages under N3C5 and N2C5 were higher than those under the N3CF treatment.

### 2.5. Leaf Area Index

**Figure 6:**
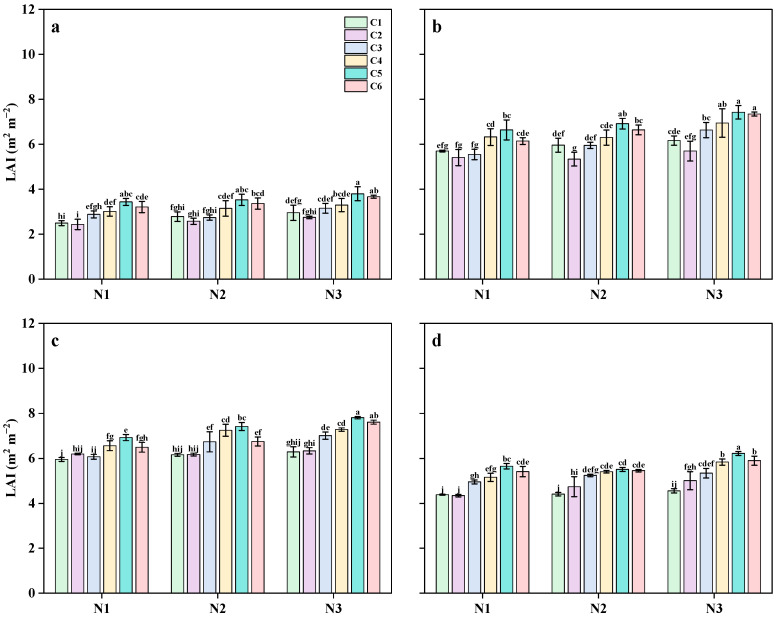
Effects of different slow-release fertilizer ratios at different N application rates on leaf area index at MT (**a**), PI (**b**), HD (**c**) and FS (**d**) of rice in 2021. Note: Data are means of replicates, and the vertical bars represent the standard error. Different letters above the columns indicate statistical significance at the *p* < 0.05 level for different treatments. N1, 90 kg N ha^−1^; N2, 120 kg N ha^−1^; N3, 150 kg N ha^−1^; C1, sulfur-coated urea (SCU): resin-coated urea (RCU) = 1:0; C2, SCU: RCU = 0:1; C3, SCU: RCU = 3:1; C4, SCU: RCU = 4:1; C5, SCU: RCU = 5:1; C6, SCU: RCU = 6:1.

**Figure 7:**
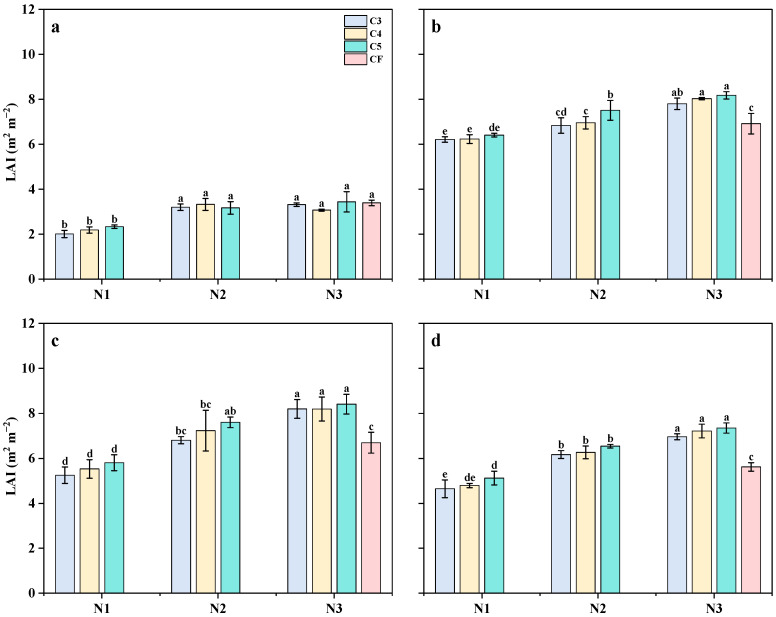
Effects of different slow-release fertilizer ratios at different N application rates on leaf area index at MT (**a**), PI (**b**), HD (**c**) and FS (**d**) of rice in 2022. Note: Data are means of replicates, and the vertical bars represent the standard error. Different letters above the columns indicate statistical significance at the *p* < 0.05 level for different treatments. N1, 90 kg N ha^−1^; N2, 120 kg N ha^−1^; N3, 150 kg N ha^−1^; C3, sulfur-coated urea (SCU): resin-coated urea (RCU) = 3:1; C4, SCU: RCU = 4:1; C5, SCU: RCU = 5:1.

The LAI of MT, PI, HD, and HS reached its maximum at the HD stage as the growth period progressed and began to decline at the HS stage. The LAI at different growth stages increased with higher N application rates (Figure 6 and Figure 7). Compared with the N1 treatment, the LAI of the N2 treatment increased by 22.9%, 8.5%, 17.4%, and 16.4% at the MT, PI, HD, and FS stages, respectively, while the N3 treatment increased by 28.6%, 20.1%, 28.8%, and 28.6% at the same stages. The LAI of each period increased with the increase in N application rate. The LAI of single SCU and RCU applications was lower than that of the combined application of the two fertilizers, except during the PI stage. In the early growth stage, the LAI of the C2 treatment was lower than that of the C1 treatment. However, in the late growth stage, the LAI of the C2 treatment was slightly higher than that of the C1 treatment. Regarding the ratio of slow- and controlled-release fertilizers, the LAI of rice under single applications of SCU (C1) and RCU (C2) was lower than that of the two fertilizers. As the SCU:RCU ratio increased from 3:1 to 5:1, the LAI exhibited an upward trend. The LAI at each growth stage under C4 treatment was 4.3%, 4.6%, 4.9%, and 4.1% higher than that of the C3 treatment. Under C5 treatment, the LAI at each stage was 18.4%, 9.8%, 9.1%, and 7.9% higher than that of C3 treatment. When the SCU:RCU ratio increased further from 5:1 to 6:1, the LAI showed a downward trend. Overall, the LAI of N3C5 and N2C5 at each growth stage was higher than that of the N3CF treatment.

### 2.6. Nitrogen Use Efficiency

Total N absorption at HD and PM stages increased with the increase in N application rate and the proportion of SCU in the slow-release fertilizer ratio. The ratio of N fertilizer and slow-controlled-release fertilizer had significant impacts on TNHD, TNPM, NFP, NHI, NAUE, and NPE. On the other hand, the ratio of N fertilizer and slow-controlled-release fertilizer had no significant interaction on TNHD, TNPM, NFP, NAE, NHI, NAUE, and NPE. NFP and NPE decreased with the increase in N application rate, whereas NAE and NAUE increased with the increase in N application rate (Table 3). With the increase in the proportion of SCU in the slow-release fertilizer ratio, NFP, NAE, and NAUE increased, while NHI and NPE decreased. Compared with N3CF, TNHD, TNPM, NFP, NHI, NAE, and NAUE under the N2C5 treatment increased by 6.4%, 15.0%, 26.6%, 4.0%, 28.1%, and 55.9%, respectively. Under the N3C5 treatment, TNHD, TNPM, NFP, NHI, NAE, and NAUE increased by 27.9%, 32.2%, 9.5%, 2.6%, 17.6%, and 53.2%, respectively, compared with N3CF.

### 2.7. Effects of Dry Matter Accumulation and LAI on Yield, Photosynthetic Rate and NUE

**Figure 8:**
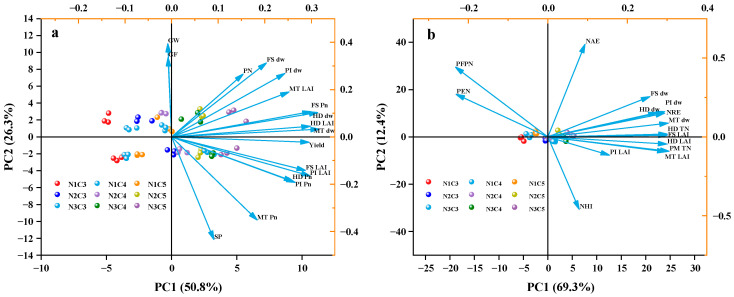
Principal Component Analysis of Biomass Accumulation, LAI, and Yield Components (**a**) and Biomass Accumulation, LAI, and N Use Efficiency (**b**) under Different N Application Rates and Controlled-Release Fertilizer Ratios. Note: DW, dry weight; GW, Grain Weight; SP, Spikelets Panicle^−1^; GF, Grain Filling; PN, Panicles Number; Pn, Net photosynthetic rate; MT, mid-tillering stage; PI, panicle initiation stage; HD, heading stage; FS, filling stage; PM, physiogical maturity stage; NHI, N harvest index; NFP, N fertilizer productivity; NPE, N Physiological Utilization Efficiency; NAUE, N Apparent use efficiency; NAE, N Agricultural utilization efficiency; TN, Total N uptake.

Principal component analysis (PCA) was conducted to assess the relative contribution of photosynthetic traits, material accumulation, and NUE to crop yield, considering three N levels and three ratios of slow-release compound fertilizer (Figure 8). The results indicated that the second and third PCA axes accounted for over 80% of the total variation observed in rice (Figure 8). Correlation analysis further revealed significant correlations among photosynthetic traits, material accumulation, and NUE in rice (Figure 8). Among the indicators related to photosynthetic traits, dry matter accumulation at the MT stage showed the strongest correlation with crop yield (Figure 8). Specifically, dry matter accumulation of rice at MT was significantly and positively correlated with crop yield.

**Figure 9:**
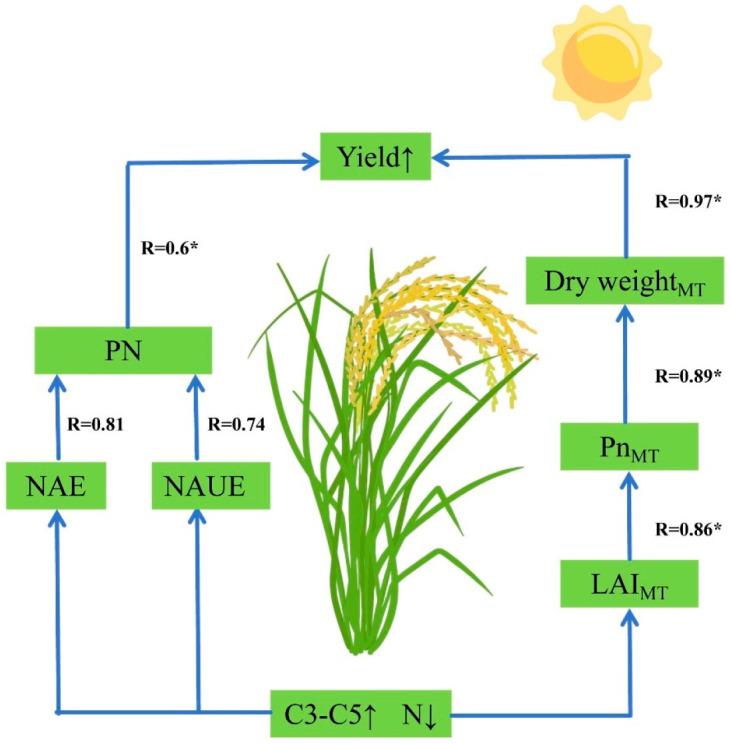
Mechanism of the Effect of Increasing the Proportion of Sulfur-Coated Urea in Slow-Release Compound Fertilizer and Reducing N Application Rate on Rice Yield. Note: In ANOVA, * indicate significance at 0.05 probability levels. “↑” indicates an increase, while “↓” indicates a decrease. MT, mid-tillering stage; PN, Panicles Number; NAUE, N Apparent use efficiency; NAE, N Agricultural utilization efficiency; Pn, Net photosynthetic rate; LAI, Leaf Area Index; C3, sulfur-coated urea (SCU): resin-coated urea (RCU) = 3:1; C4, SCU: RCU = 4:1; C5, SCU: RCU = 5:1.

By increasing the proportion of SCU in slow-release compound fertilizers and reducing the N application rate in N fertilizer management measures, crop yield can be significantly increased. From the perspective of yield components, this N management strategy increased both NAE and NAUE, which in turn increased the number of effective panicles and thus increased yield. From the perspective of material production, our N management measures enhanced the photosynthetic rate by increasing the LAI during each growth stage (particularly the MT stage), leading to greater dry matter accumulation (Figure 9). This higher dry matter accumulation was strongly correlated with increased yield (R = 0.97).

## 3. Discussion

### 3.1. Effects of N Application Rate and Slow-Release Fertilizer Ratio on Rice Yield Formation and NUE

N application rate and fertilizer type are key factors in regulating rice population and play a crucial role in determining rice yield. In this study, increasing the N application rate from 90 kg ha^−1^ to 120 kg ha^−1^ and 150 kg ha^−1^ resulted in average yield increases of 13.1% and 20.1%, respectively. This yield improvement was mainly due to an increase in the number of effective panicles and grains per panicle. Previous studies have also shown that higher N application rates lead to increased panicle number, total spikelet number, and grain yield [22,23]. Controlled-release N fertilizers, which gradually release N, can significantly reduce N losses and improve labor efficiency. As a result, they serve as an effective strategy for enhancing fertilizer use efficiency while minimizing environmental impact. The use of controlled-release N fertilizers has been shown to increase rice yield by increasing the number of panicles per square meter in pot-seedling machine-transplanted rice [24]. Wang et al. [25] reported that the use of controlled-release urea increased yields by 10.8% and 5.6% compared with farmers’ traditional fertilization practices and improved fertilization methods, respectively. The main reason for the increase in grain yield by controlled-release N fertilizer treatment was to increase the number of spikes and spikelets per square meter. Moreover, Yu et al. [26] found that a single basal application of 70% controlled-release urea blended with 30% conventional urea increased rice yield by 25.6% compared with split applications of full conventional urea. However, the rapid N release from SCU during early growth stages can lead to insufficient N availability later in the rice growth cycle, ultimately reducing both grain yield and panicle number at maturity. This aligns with our research findings (Figure 1, Table 1 and Table 2). After adjusting the ratio of compound slow-release N fertilizer, increasing the SCU:RCU ratio from 3:1 to 5:1 significantly increased rice yield (Figure 1). Applying a single slow-release fertilizer at the basal stage may be insufficient to meet the rice’s N demand throughout its entire growth period. Due to their delayed N availability, controlled-release fertilizers are often combined with conventional urea to address the early N requirements of rice [27]. Basal application of sulfur-coated/controlled-release fertilizer, supplemented with conventional urea at the tillering stage, can enhance the formation of effective tillers and spikelets [20], aligning with the rationale for adopting composite controlled-release N fertilizer in this study. Increasing the SCU to RCU ratio from 3:1 to 5:1 significantly increased the number of effective panicles (Table 1). Furthermore, at application rates of 120 kg N ha^−1^ and 150 kg N ha^−1^, the 5:1 controlled-release fertilizer ratio increased the number of effective panicles by 6.7% and 11.1%, respectively, compared to traditional split fertilization applied at 150 kg N ha^−1^ (Table 2 and Table 3). The yield improvement observed under the 5:1 slow-release fertilizer ratio can be attributed to its higher proportion of sulfur-coated urea, which enhanced N availability during the early growth stage. This adequate N supply during the tillering phase promoted tiller development, increasing the number of tillers per plant and thus resulting in more effective panicles at maturity.

From the perspective of N absorption and utilization, rice yield can be expressed as the product of total N uptake by plants at maturity and the N production efficiency of grains. Enhancing NUE is one of the main research directions to achieve both high yield and high efficiency in rice cultivation. It has been reported [28] that N application increases N accumulation in rice but leads to a decrease in NUE. Our results also indicate that increasing the N application rate can increase total N uptake at HD and PM but reduce NUE. The NPE of the N2 and N3 treatments was 13.9% and 17.0% lower than that of the N1 treatment, respectively. Excessive use of N fertilizers can result in overgrowth of roots. A reasonable N application rate can prevent excessive root growth, transport N to the aboveground parts, and ensure efficient use of N fertilizer while maintaining yield [29]. Under flooded conditions, urea undergoes rapid hydrolysis, and N migration occurs quickly. Although adequate N availability during early growth stages promotes vigorous tillering, it can also lead to the production of numerous ineffective tillers. Some of the applied N becomes immobilized in these nonproductive tillers, limiting its availability for grain formation. Additionally, an extremely dense population and “flourishing branches and lush leaves” had a negative impact on N transport and distribution. During the reproductive growth stage, as ineffective tillers disappeared, N was transported to the panicle to maintain the increase in N in the panicle. Slow/controlled-release fertilizer release pattern was less affected by water, maintaining higher N availability, which helped preserve the spike rate to a certain extent and improved N use efficiency [30]. Studies on rice have shown that the application of controlled-release N fertilizers can enhance root vitality, thereby improving N uptake [9]. Therefore, applying slow-release fertilizer can also delay root senescence during the late growth stages of rice and promote the absorption and utilization of soil N by roots, allowing rice to absorb more N from PI to PM. In this study, increasing the ratio of SCU to RCU to 5:1 proved to be the optimal slow-release fertilizer ratio, significantly increasing TNHD, TNPM, NFP, NAE, and NAUE (Table 3). Compared with the traditional 150 N split fertilization, the NUE indexes were higher than those of the traditional split fertilization when the SCU:RCU ratio was 5:1 under both 150 N and 120 N applications.

Therefore, an appropriate N application rate combined with a suitable slow-release fertilizer ratio promotes the development of an excellent population structure, which increases the number of effective panicles and grains per panicle. This, in turn, increases dry matter accumulation, total N absorption, and NUE, ultimately increasing yield (Figure 8 and Figure 9). Consequently, the synergistic improvement of yield and resource utilization efficiency can be achieved through reasonable cultivation control measures.

### 3.2. The Effect of N Application Rate and Controlled-Release Fertilizer Ratio on Rice Material Accumulation and Photosynthetic Traits

Photosynthesis plays a crucial role in determining crop yield, providing nearly 70% of the material required for crop yield [31]. The production of crop dry matter mainly originates from photosynthesis. Studies have shown that enhancing the photosynthetic capacity of rice leaves is essential for increasing dry matter accumulation [32]. Improvements in rice grain yield are closely associated with changes in photosynthetic traits [33]. Consequently, suitable LAI, strong photosynthetic matter production, and efficient dry matter accumulation form the basis for achieving high rice yields [34,35]. Previous studies have demonstrated that leaf photosynthesis is the main driver of assimilate accumulation and partitioning in plants. The continuous function of leaves during the FS is the basis for promoting photosynthetic performance and assimilation accumulation, which largely determines grain weight and overall yield [36]. Appropriate N application enhances leaf photosynthetic capacity by increasing LAI, boosting chlorophyll content, and delaying leaf senescence [25]. In this study, the LAI, dry matter accumulation, and net photosynthetic rate of rice increased significantly when the N application rate increased from 90 kg ha^−1^ to 120 and 150 kg ha^−1^. These findings align with those of Qadeer et al. [37] and are consistent with the results of Abbas et al. [38]. Liu et al. [39] also reported that both dry matter accumulation and light use efficiency showed a positive correlation with N application rates. Similar to this, our results indicated that mean dry matter accumulation under the N2 and N3 treatments yielded 15 and 16.2 t ha^−1^, respectively, corresponding to increases of 15.0% and 24.1% compared with the N1 treatment (Figure 2). In this study, the application of slow-release fertilizer significantly increased the dry matter accumulation, LAI, and photosynthetic rate of rice compared with traditional split fertilization. At the N3 level, increasing the proportion of SCU in slow-release compound fertilizer significantly increases dry matter accumulation, LAI, and photosynthetic rate of rice. This is due to the fact that the fertilization method and N fertilizer structure are more reasonable through N fertilizer management measures. These adjustments are in line with the N demand of rice throughout its growth period. Consequently, the maintenance time of green leaf area becomes longer, and the degradation of leaf area after flowering becomes slower. By delaying leaf senescence and prolonging photosynthetic time, grain yield is increased [40].

Li et al. [41] reported that, compared to conventional urea, controlled-release urea significantly increased the net photosynthetic rate of flag leaves during the mid- and late-growth stages of rice. This extended the photosynthetic activity of functional leaves, delayed senescence, and ultimately improved grain yield. This experiment showed that increasing both the N application rate and the proportion of SCU in controlled-release fertilizers significantly enhanced the LAI, net photosynthetic rate, and dry matter accumulation in rice. By supplying sufficient N during the early growth stage, the C5 treatment increased the leaf area index of rice, which helped delay leaf senescence and improve photosynthetic performance. The use of controlled-release fertilizers has been shown to increase the LAI in hand-cultivated rice [42], contributing to higher dry matter production [43]. Moreover, the application of slow- and controlled-release fertilizers significantly increased the intrinsic water-use efficiency of rice at the photosynthetic level, which may enhance nutrient transport in plants [44]. Controlled-release fertilizers delay N release, thereby prolonging the nutrient availability period and enhancing the synchronization between the N demand of rice plants and the N supply from the fertilizer. The study of Pu et al. [45] indicated that mechanized deep application of slow/controlled-release fertilizers had a greater effect on grain yield than urea or compound fertilizer. This is largely because the number of effective panicles per hectare and the number of spikelets per panicle increased. The reason for this increase may be that mechanized deep application of slow/controlled-release fertilizers promoted rice growth, such as relatively high LAI and total aboveground biomass.

### 3.3. Optimizing the Ratio of Controlled-Release Fertilizers to Enhance Rice Photosynthetic Capacity and NUE

The photosynthetic rate is widely regarded as one of the most reliable physiological indicators of crop growth and productivity [46]. It is positively correlated with NUE, as N is a major component of essential photosynthetic enzymes, such as Rubisco. In recent years, slow-release fertilizers have been proposed to improve crop NUE because they can consistently meet crop N requirements throughout the growing season, thereby promoting higher crop yields [47]. Controlled-release fertilizers delay N release and prolong nutrient availability. This improves the synchronization between the N demand of rice plants and the N supply from the fertilizer, ultimately boosting yields (Figure 1a). Leaves serve as the primary sites of photosynthesis and largely determine dry matter production and grain yield in grain crops [48]. N, as a basic component of biomolecules (e.g., chlorophyll and leaf enzymes), is closely linked to photosynthetic capacity and dry matter production of rice plants [49]. Appropriate N application is known to enhance leaf photosynthetic capacity by increasing LAI and chlorophyll content while also delaying leaf senescence [25,37,38]. In this study, optimizing the ratio of slow- and controlled-release fertilizers at the same N level significantly increased both LAI and photosynthetic rate. Thus, optimizing the ratio of controlled-release fertilizers improved canopy photosynthetic capacity, enhanced leaf photosynthetic rates, and ultimately increased grain yield in rice. Our study found that optimizing the ratio of slow-release fertilizer (SCU: RCU = 5:1) at the same N level mainly increased the number of effective panicles and the apparent utilization rate of N fertilizer, thereby increasing grain yield. The effect on the effective panicle number was more pronounced in the MT. The application of slow-release compound fertilizer (SCU: RCU = 5:1) at the MT increased the LAI, net photosynthetic rate, and dry matter accumulation of rice. This allowed rice at the MT to obtain sufficient N early in growth due to the fast-release characteristics of SCU, promoting early-stage tillering. Peng et al. [50] demonstrated that controlled-release fertilizers enhance rice yield by increasing both panicle number and spikelet number per panicle. Our results are consistent with these findings. This study shows that optimizing the controlled-release fertilizer ratio (N2C5) while reducing the overall N application rate leads to higher grain yield, improved NUE, and enhanced photosynthetic rates compared to conventional split fertilization (N3CF). These results indicate that refining controlled-release fertilizer formulations to reduce N input can effectively unlock the potential for high-yield and high-efficiency rice production. However, the N fertilizer gradient in this trial was relatively limited. In addition, the specific optimal N application rate and controlled-release fertilizer ratio require further experimental investigation. Our main focus in this trial was on the photosynthetic activity and material accumulation in the aboveground parts of rice plants. Future studies should place greater emphasis on optimizing the effects of controlled-release fertilizers on both the soil environment and plant root systems.

## 4. Materials and Methods

### 4.1. Experiment Site

The two-year experiment (2021–2022) was conducted at the National Agricultural Variety Testing and Characterization Station, Jilin Agricultural University, Changchun City, Jilin Province (44°46′ N, 125°39′ E), China. This site is located in the Northeast Songliao Plain, which is characterized by a temperate continental humid climate. During the rice growth period, the accumulated temperatures in 2021 and 2022 were 2991 °C and 2800 °C, respectively, with total rainfall of 860.3 mm and 600 mm, respectively. The average basic physicochemical properties of the 0–20 cm soil layer over the two years were organic carbon content 9.60 g kg^−1^, alkali-hydrolyzable N content 33.89 mg kg^−1^, available potassium content 137.09 mg kg^−1^, available phosphorus content 29.42 mg kg^−1^, and pH 6.70.

### 4.2. Experiment Design

This experiment employed a randomized block design using Jinongda 667 as the experimental material. In 2021, eighteen treatments were established, each with three replicates. Individual plot areas measured 30 m^2^. Three N fertilizer gradients were set: 90 kg ha^−1^ controlled-release fertilizer (N1), 120 kg ha^−1^ controlled-release fertilizer (N2), and 150 kg ha^−1^ controlled-release fertilizer (N3). Additionally, six controlled-release fertilizer ratios were established: SCU: RCU = 1:0 (C1), 0:1 (C2), 3:1 (C3), 4:1 (C4), 5:1 (C5), and 6:1 (C6) for controlled-release fertilizer ratios. Sulfur-coated urea is a sulfur-coated urea fertilizer produced by Anshan Shunda Compound Fertilizer Factory, featuring an “S”-shaped release curve with a release duration of ≥90 days. Resin-coated urea is a resin-coated urea produced by Changchun Jinnongda Agricultural Technology Co., Ltd., exhibiting an “S”-shaped release curve with a release duration of ≥90 days. All controlled-release fertilizers were used as basal fertilizer and incorporated into the soil at a depth of 10–15 cm using rotary tillage. For CF treatments, urea was applied in a split regimen at a basal/tillering/panicle ratio of 6:3:1. Phosphorus (as P_2_O_5_) and potassium (as K_2_O) fertilizers were applied as basal dressings at rates of 98 kg ha^−1^ and 75 kg ha^−1^, respectively. In 2022, eleven treatments were established based on the 2021 experimental design. The three N fertilizer gradients were maintained from the previous year: 90 kg ha^−1^ (N1), 120 kg ha^−1^ (N2), and 150 kg ha^−1^ (N3) of controlled-release fertilizer. In addition, three ratios of SCU to RCU were retained: 3:1 (C3), 4:1 (C4), and 5:1 (C5), with controlled-release fertilizer ratios being partially optimized. The application rates for phosphorus and potassium fertilizers, as well as the application methods for controlled-release fertilizers, remained consistent with those used in 2021. At the same time, a control treatment without N fertilizer application (CK) and a traditional split application of N fertilizer at 150 kg ha^−1^ were established. The traditional N fertilizer application was denoted as CF.

Rice seedlings were sown on 15 April 2021, and hand-transplanted on 20 May at the three-leaf stage. Transplanting density was maintained at 30 cm × 13 cm spacing with three seedlings per hill. The final harvest was conducted on 28 September. In 2022, rice seedlings were sown on 13 April and hand-transplanted at the three-leaf stage on 25 May. Transplanting density was set at 30 cm × 13 cm with five seedlings per hill. Independent irrigation and drainage systems were installed for each experimental plot. All other field management practices followed regionally recommended high-yielding protocols. The final harvest took place on 1 October.

### 4.3. Sampling and Measurements

#### 4.3.1. Grain Yield and Yield Components

Three survey points were selected in each plot at the physiological maturity stage. The effective panicles of 25 holes were examined at each survey point. Based on the average number of effective panicles, 10 representative rice plants were selected. After natural air drying, the number of grains per panicle, 1000-grain weight, and seed-setting rate were measured. During the mature period, five 1 m^2^ were selected from each plot for yield measurement, and the yield was calculated. After drying, the weight of the rice grains was recorded, and the moisture content was determined using a grain moisture meter. The rice yield was then calculated based on a standard moisture content of 13.5%.

#### 4.3.2. Dry Matter Accumulation

Five representative plants with uniform growth vigor were selected from each plot at MT, PI, HD, FS, and PM. After removing the roots, the plants were separated into stems, leaves, and panicles. The plant samples were deactivated at 105 °C for 30 min, then dried at 80 °C until reaching constant weight. Finally, the dry matter weight was measured using a ten-thousandth balance.

#### 4.3.3. N Accumulation and N Fertilizer Utilization Efficiency

Samples were collected during the rice maturation period. Five plants were selected from each plot, and their stems, leaves, and panicles were separated. The samples were then subjected to 105 °C heat treatment for 30 min, followed by drying at 80 °C until a constant weight was achieved. After grinding, the samples were digested using H_2_SO_4_-H_2_O_2_. Finally, the N content of each organ sample was determined using an automatic Kjeldahl N analyzer. The calculation formula is as follows:

Total N fertilizer uptake (kg ha^−1^) = Above-ground dry matter mass at maturity × N content

N Agricultural utilization efficiency (NAE) (kg kg^−1^) = (Yield in N-applied area − Yield in N-free area)/N fertilizer application rate

N Apparent Use Efficiency (NAUE) (%) = (Total N uptake by plants in the N-applied area − Total N uptake by plants in the N-free area)/N fertilizer application rate × 100%

N fertilizer productivity (NFP) (kg kg^−1^) = Yield in the N-applied area/N fertilizer application rate

N Harvest Index (NHI) (%) = Grain N accumulation at harvest/Total plant N accumulation

N Physiological Utilization Efficiency (NPE) (kg kg^−1^) = (N-applied zone yield − N-free zone yield)/(Total N uptake by plants in the N-applied zone − Total N uptake by plants in the N-free zone)

#### 4.3.4. Net Photosynthetic Rate

Net photosynthetic rates were measured during the MT, PI, HD, and FS stages. Measurements were taken on sunny, windless mornings between 9:00 and 11:00 a.m. A portable photosynthesis meter (LI-6400 model, LI-COR, Lincoln, NE, USA) was used to determine the net photosynthetic rate of rice flag leaves, with five replicates per treatment.

#### 4.3.5. LAI

During the MT, PI, HD, and FS stages, five representative plants with uniform growth were selected from each plot. Leaf area was measured using the length × width coefficient method and then converted into LAI using the following formula:

LAI = (Leaf Shape Factor × Sum of Leaf Length × Width)/Land Area

### 4.4. Statistical Analysis

Statistical analysis was conducted using IBM SPSS Statistics 26 (IBM SPSS Inc., Somers, NY, USA) software. One-way analysis of variance (ANOVA) was conducted, and Duncan’s method was used for multiple comparisons. Differences between means were considered statistically significant at the level of <0.05. The mean values with standard errors (SE) were reported for all data. Figures were generated using Origin 2023 (Origin Lab, Corporation, Northampton, MA, USA) software. Data organization and table generation were carried out in Microsoft Excel 2021.

## 5. Conclusions

Increasing N fertilizer increased yield but reduced NUE. However, adjusting the slow-controlled fertilization ratio can save labor and enhance fertilizer use efficiency while maintaining high yield by increasing dry matter accumulation, LAI, photosynthetic rate, and NUE. By increasing the proportion of SCU in the slow-release compound fertilizer, early-stage N deficiency in rice growth was alleviated, leaf senescence was delayed, and the transport of N from roots to stems and leaves in the early stage was ensured. This stable nutrient supply supported rice tillering, resulting in enough effective panicles during the late stage of rice growth and ultimately leading to significant improvements in both NUE and yield. Although the N3C5 treatment performed best in all aspects, the N2C5 treatment is more suitable for achieving efficient fertilizer use. It meets the production requirements for high-yield and high-efficiency rice while also reducing production costs. There is a close relationship between yield, photosynthetic traits, and NUE. Increasing the proportion of SCU in slow-release compound fertilizer, along with reasonable regulation of fertilizer management measures and reduced N application rates, can synergistically improve rice yield, photosynthetic rate, and NUE.

## Figures and Tables

**Table 1:** Effect of slow-release fertilizer ratios on rice yield components under different N application rates in 2021.

Nitrogen	Slow-Release Fertilizer Ratios	Spikelets Panicle^−1^	Grain Filling (%)	Grain Weight (g Per 1000 Seeds)	Panicles Number (m^2^)
N1	C1	97.6 e	94.3 bc	25.1 a	241.3 i
	C2	102.0 de	94.9 abc	23.8 abcd	289.0 gh
	C3	102.4 de	96.6 abc	24.5 abc	303.3 fg
	C4	103.8 de	95.8 abc	23.4 abcd	338.0 def
	C5	118.2 bc	94.4 bc	22.3 d	474.0 b
	C6	102.5 de	95.3 abc	23.5 abcd	310.0 efg
N2	C1	103.8 de	93.4 c	22.8 cd	263.0 hi
	C2	102.0 de	87.4 d	22.3 d	340.0 def
	C3	102.5 de	95.5 abc	23.6 abcd	376.0 cd
	C4	115.3 bc	97.8 ab	24.3 abc	377.3 cd
	C5	119.6 b	95.5 abc	23.9 abcd	402.3 c
	C6	103.8 de	98.2 a	23.1 bcd	347.7 de
N3	C1	101.8 de	96.8 ab	22.7 cd	299.0 fgh
	C2	109.7 cd	95.1 abc	23.7 abcd	347.7 de
	C3	112.5 bc	94.5 bc	23.7 abcd	375.3 cd
	C4	114.3 bc	96.0 abc	23.9 abcd	397.3 c
	C5	141.6 a	96.9 ab	23.9 abcd	515.8 a
	C6	103.8 de	94.7 bc	24.7 ab	471.3 b
Analysis of variance					
N		**	NS	NS	**
C		**	**	NS	**
N×C		**	**	*	**

Note: Data are means of replicates. Different letters following the columns indicate statistically significant differences at the *p* < 0.05 level. In ANOVA, * and ** indicate significance at 0.05 and 0.01 probability levels, respectively, while NS indicates no significant difference. N1, 90 kg N ha^−1^; N2, 120 kg N ha^−1^; N3, 150 kg N ha^−1^; C1, sulfur-coated urea (SCU): resin-coated urea (RCU) = 1:0; C2, SCU: RCU = 0:1; C3, SCU: RCU = 3:1; C4, SCU: RCU = 4:1; C5, SCU: RCU = 5:1; C6, SCU: RCU = 6:1.

**Table 2:** Effect of slow-release fertilizer ratios on rice yield components under different N application rates in 2022.

Nitrogen	Slow-Release Fertilizer Ratios	Spikelets Panicle^−1^	Grain Filling (%)	Grain Weight (g per 1000 Seeds)	Panicles Number (m^2^)
N1	C3	139.2 cd	93.8 ab	21.5 b	258.0 f
	C4	137.7 d	94.2 ab	21.5 b	267.0 ef
	C5	137.7 d	94.3 ab	21.6 b	275.7 de
N2	C3	139.6 cd	94.8 a	21.9 ab	292.0 cd
	C4	140.8 bcd	94.3 ab	21.9 ab	299.3 bc
	C5	146.1 ab	93.9 ab	21.8 ab	309.3 ab
N3	C3	144.9 ab	94.7 ab	22.1 ab	315.0 ab
	C4	145.7 ab	93.8 ab	22.1 ab	318.6 a
	C5	148.7 a	94.6 ab	22.0 ab	322.2 a
	CF	290.0 cd	146.7 a	22.9 a	146.7 a
CK		168.3 g	121.9 e	22.0 ab	121.9 e
Analysis of variance					
N C N×C		**	NS	NS	**
		NS	NS	NS	**
		NS	NS	NS	NS

Note: Data are means of replicates. Different letters following the columns indicate statistically significant differences at the *p* < 0.05 level. In ANOVA, ** indicate significance at 0.01 probability levels, respectively, while NS indicates no significant difference. N1, 90 kg N ha^−1^; N2, 120 kg N ha^−1^; N3, 150 kg N ha^−1^; C3, sulfur-coated urea (SCU): resin-coated urea (RCU) = 3:1; C4, SCU: RCU = 4:1; C5, SCU: RCU = 5:1; CF, traditional split-application fertilization; CK, without N fertilizer application.

**Table 3:** Effects of slow-release fertilizer ratios on N accumulation and NUE in rice under different N application rates in 2022.

Nitrogen	Slow-Release Fertilizer Ratios	TN HD (kg ha^−1^)	TN PM (kg ha^−1^)	NFP (kg kg^−1^)	NHI (%)	NAE (kg kg^−1^)	NAUE (%)	NPE (kg kg^−1^)
N1	C3	49.95 i	69.12 i	80.46 bc	72.79 abc	33.42 cd	37.40 d	89.68 a
	C4	60.42 h	74.64 h	82.25 ab	70.30 d	35.82 bcd	43.53 c	82.57 ab
	C5	65.09 g	78.81 g	86.00 a	69.82 d	38.96 ab	48.17 b	79.74 abc
N2	C3	68.49 g	93.98 f	70.31 ef	73.69 a	35.03 bcd	48.77 b	71.91 bc
	C4	72.94 f	98.01 e	72.47 de	73.72 a	37.19 bcd	52.13 ab	71.44 bc
	C5	81.71 d	103.43 d	77.02 cd	72.63 abc	41.75 a	56.64 a	73.71 bc
N3	C3	87.09 c	109.35 c	63.75 gh	73.41 ab	35.53 bcd	49.43 b	71.88 bc
	C4	92.45 b	114.51 b	64.18 gh	72.18 bc	35.96 bcd	52.70 ab	68.41 c
	C5	98.26 a	118.91 a	66.56 fg	71.61 c	38.34 abc	55.66 a	68.93 c
	CF	76.82 e	89.96 f	60.82 h	69.81 d	32.60 d	36.34 d	90.08 a
CK		26.07 j	35.46 j	——	——	——	——	——
Analysis of variance								
N		**	**	**	**	NS	**	**
C		**	**	**	**	**	**	NS
N×C		NS	NS	NS	NS	NS	NS	NS

Note: Data are means of replicates, and the vertical bars represent the standard error. Different letters following the columns indicate statistically significant differences at the *p* < 0.05 level. In ANOVA, ** indicate significance at 0.01 probability levels, respectively, while NS indicates no significant difference. N1, 90 kg N ha^−1^; N2, 120 kg N ha^−1^; N3, 150 kg N ha^−1^; C3, sulfur-coated urea (SCU): resin-coated urea (RCU) = 3:1; C4, SCU: RCU = 4:1; C5, SCU: RCU = 5:1; CF, traditional split-application fertilization. TN HD, Total N uptake in heading stage; TN PM, Total N uptake in physiological maturity stage; NHI, N harvest index; NFP, N fertilizer productivity; NPE, N Physiological Utilization Efficiency; NAUE, N Apparent fertilizer utilization rate; NAE, N Agricultural utilization efficiency.

## Data Availability

The data presented in this study are available on request from the corresponding author. The data are not publicly available due to privacy and ethical restrictions.
